# Systemic inflammatory biomarkers linked to *PKD1* mutation and renal morphometrics in cats with polycystic kidney disease

**DOI:** 10.14202/vetworld.2026.1495-1503

**Published:** 2026-04-24

**Authors:** Kotchapol Jaturanratsamee, Palin Jiwaganont, Wannisa Meepoo, Ratikorn Bootcha, Soontaree Petchdee

**Affiliations:** 1 Graduate School, Science and Innovation for Animal Health Program, Faculty of Veterinary Medicine, Kasetsart University, Thailand; 2 Kasetsart University Veterinary Teaching Hospital, Faculty of Veterinary Medicine, Kasetsart University, Kamphaeng Saen, Nakorn Pathom, Thailand; 3 Department of Large Animal and Wildlife Clinical Sciences, Faculty of Veterinary Medicine, Kasetsart University, Kamphaeng Saen, Nakorn Pathom, Thailand

**Keywords:** biomarkers, feline polycystic kidney disease, inflammation, neutrophil-to-lymphocyte ratio, pkd1 mutation, renal morphometrics, Scottish Fold cats, ultrasonography

## Abstract

**Background and Aim:**

Feline polycystic kidney disease (PKD) is the most common inherited renal disorder in cats and is primarily associated with a nonsense mutation in the *PKD1* gene. Although ultrasonography and genetic testing are well-established diagnostic tools, little is known about systemic inflammatory changes during the subclinical stages of feline PKD. This study aimed to evaluate the association between inflammatory biomarkers, *PKD1* mutation status, and renal morphometric changes in cats with polycystic kidney disease.

**Materials and Methods:**

This retrospective, single-center observational study included 28 client-owned cats evaluated by renal ultrasonography and *PKD1* genotyping. Based on imaging findings and genetic status, cats were classified into three groups: healthy controls without renal cysts or *PKD1* mutation, cats with renal cysts and wild-type *PKD1*, and cats with renal cysts carrying a heterozygous *PKD1* mutation. Hematological inflammatory indices, including neutrophil-to-lymphocyte ratio (NLR), monocyte-to-lymphocyte ratio (MLR), and platelet-to-lymphocyte ratio (PLR), were calculated from routine blood profiles. Conventional renal biomarkers (creatinine, blood urea nitrogen, and symmetric dimethylarginine) and ultrasonographic renal morphometric parameters were also assessed. Correlations between inflammatory markers, renal measurements, biochemical indices, and genotype were analyzed using Pearson’s correlation.

**Results:**

All cats were clinically stable and non-azotemic at the time of evaluation. Conventional renal biomarkers did not differ significantly among groups. However, cats with renal cysts showed stronger associations between inflammatory indices and renal morphometric parameters compared with healthy controls. These correlations were most pronounced in cats harboring a heterozygous *PKD1* mutation, where NLR and MLR were positively associated with kidney size and renal biochemical markers despite values remaining within reference ranges. Scottish Fold cats showed a high prevalence of *PKD1* heterozygosity. Overall, systemic inflammatory indices demonstrated closer relationships with renal structure and function than traditional renal biomarkers during early disease stages.

**Conclusion:**

Systemic inflammatory activation is detectable in cats with PKD before the onset of azotemia, particularly in those carrying *PKD1* mutations. Simple hematological inflammatory ratios may serve as accessible adjunct tools alongside ultrasonography and genetic testing for the early detection and monitoring of feline PKD. Further longitudinal studies are warranted to validate their prognostic utility.

## INTRODUCTION

Cats have become increasingly popular companion animals, and advances in feline medicine have consequently placed greater emphasis on inherited disorders [1]. Polycystic kidney disease (PKD) is among the most common inherited renal disorders in cats and is characterized by the progressive development of multiple renal cysts in the absence of other underlying causes, such as elevated symmetric dimethylarginine (SDMA) [2–4]. Feline PKD is most frequently reported in Persian and Persian-related breeds and is inherited as an autosomal dominant trait [5]. The identification of causative genetic variants has substantially improved the understanding of PKD in cats. In particular, a nonsense mutation in the *PKD1* gene (*c.10063 C>A*, exon 29) has been confirmed in a large proportion of affected cats [6–8]. Despite extensive genetic and ultrasonographic characterization, there remains a paucity of information regarding inflammatory biomarkers in cats with PKD and their relationships with disease severity, genotype status, and imaging findings [9].

Biomarkers are increasingly incorporated into veterinary practice for diagnostic and prognostic purposes because they provide rapid, objective, and reproducible information [10]. Our previous study identified peptides and proteins associated with familial PKD in cats using matrix-assisted laser desorption/ionization time-of-flight (MALDI-TOF) mass spectrometry combined with peptide mass fingerprinting and three-dimensional principal component analysis [11]. In addition, recent studies have demonstrated that kidney function screening can detect early renal deterioration at stages when the glomerular filtration rate has begun to decline [12]. Nevertheless, data on inflammation-related biomarkers in feline PKD remain limited, and their clinical relevance has not been fully elucidated [13].

Although PKD has been extensively characterized using ultrasonography and *PKD1* genotyping, current diagnostic approaches primarily identify structural or genetic abnormalities rather than early pathophysiological changes. Conventional renal biomarkers, including creatinine, blood urea nitrogen, and SDMA, often remain within reference ranges until substantial nephron loss has occurred, limiting their utility for early disease detection. While proteomic approaches using MALDI-TOF have provided insights into molecular alterations associated with PKD, there is limited evidence on readily accessible systemic inflammatory biomarkers and their relationship with renal morphometrics, genotype status, and early functional changes. In particular, the temporal emergence of low-grade systemic inflammation in cats with PKD, especially in the subclinical stage and in relation to *PKD1* mutation status, remains poorly understood. This knowledge gap restricts the development of cost-effective, minimally invasive tools for early risk stratification and clinical monitoring of PKD.

The present study aimed to investigate the association between systemic inflammatory indices derived from routine hematological profiles and renal morphometric, biochemical, and genetic parameters in cats with PKD. Specifically, this study sought to evaluate whether inflammatory biomarkers are associated with renal cystic changes and *PKD1* mutation status in clinically stable, non-azotemic cats, and to determine their potential value as adjunct indicators for early detection and monitoring of PKD before overt renal dysfunction becomes apparent.

## MATERIALS AND METHODS

### Ethical approval

This study was conducted in accordance with institutional guidelines for the use of animals in research and was reviewed and approved by the Ethics Committee of Kasetsart University, Thailand, under approval number ACKU-65-VET-077. The study involved client-owned cats presented to the Kasetsart University Veterinary Teaching Hospital, Kamphaeng Saen, and was based on retrospective evaluation of medical records, renal ultrasonographic findings, hematological and biochemical data, and *PKD1* genotyping results obtained during routine clinical assessment. Written informed consent was obtained from all owners before inclusion of their animals in the study. Sample collection was limited to procedures performed as part of standard veterinary diagnostic care, including peripheral blood sampling and abdominal ultrasonography, and no additional invasive procedures were undertaken solely for research purposes. All examinations and sample handling were performed by qualified veterinary personnel in accordance with accepted standards of animal welfare and good clinical practice. Confidentiality of owner and animal information was maintained throughout the study, and all data were analyzed anonymously.

### Study period and location

This retrospective, single-center observational study was conducted at the Animal Teaching Hospital, Kamphaeng Saen, Faculty of Veterinary Medicine, Kasetsart University, Thailand. Medical records and biological samples were reviewed from cats presented between January 2024 and September 2025. The hospital functions as a referral center for companion animals in central Thailand, providing routine preventive care, diagnostic imaging, and advanced clinical services. The study workflow comprised case identification and eligibility screening, clinical and ultrasonographic assessment, laboratory and molecular analyses, and statistical evaluation.

### Animals and selection of cases

A total of 28 client-owned cats (mean age 5.34 ± 2.25 years, body weight 3.50 ± 0.85 kg) were enrolled through convenience sampling based on the availability of complete clinical records and biological samples. Written informed consent was obtained from all owners before inclusion. Cats aged ≥1 year were eligible for enrollment. Exclusion criteria included concurrent chronic systemic diseases known to influence inflammatory or renal biomarkers, such as chronic kidney disease, diabetes mellitus, epilepsy, or other severe systemic illnesses, as determined by medical history, physical examination, and laboratory findings. All cats underwent standardized clinical evaluation, including assessment of sex, breed, age, body weight, and medical history. Routine diagnostic procedures included abdominal ultrasonography and blood profile analysis. Approximately 1 mL of blood was collected aseptically from peripheral veins and processed immediately for complete blood count (CBC) and serum biochemistry using standard hospital protocols.

### Ultrasonographic examination

**Figure 1:**
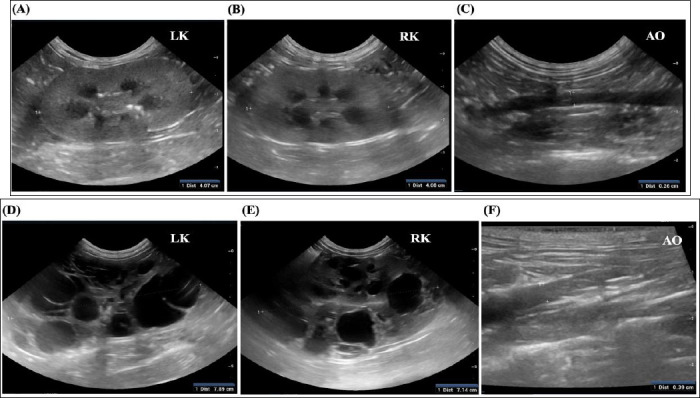
Ultrasonographic assessment of the left kidney (LK), right kidney (RK), and abdominal aorta (AO). (A–C) Control cat (Group 1). (D–F) Cat with a heterozygous *PKD1* mutation (Group 3).

Renal ultrasonography was performed by an experienced veterinary clinician using a cardiac-capable ultrasound system equipped with a high-frequency transducer. Cats were examined in right- and left-lateral recumbency following routine abdominal imaging protocols. Still images and cine loops were digitally archived for subsequent analysis. Renal cysts were defined as well-circumscribed, anechoic structures with distal acoustic enhancement. Cats presenting with more than two renal cysts were classified as having PKD according to previously published criteria [14]. All measurements were obtained from recorded images, as illustrated in Figure 1.

### DNA extraction and polymerase chain reaction

Genomic DNA was extracted from frozen whole-blood samples using a commercial DNA extraction kit according to the manufacturer’s instructions. Detection of the *PKD1* mutation was performed using polymerase chain reaction (PCR) as previously described. The primer sequences were *5'-AGCCTTCAGCAAGAAGCCA-3'* (forward) and *5'-CAAACTTGACCTTGGAGGAGC-3'* (reverse). PCR amplification followed standardized cycling conditions reported in earlier studies investigating feline PKD mutations.

### DNA sequencing

PCR products were purified using a commercial PCR purification kit before sequencing. Purified amplicons were subjected to Sanger sequencing using the same primer pairs as those used for PCR. Sequence chromatograms were analyzed and aligned using BioEdit software, and polymorphisms were identified by comparison with reference *PKD1* sequences.

### Serum biomarker analysis

Serum samples were centrifuged and stored under appropriate conditions until analysis. Hematological data obtained from the CBC were used to calculate inflammatory indices, including neutrophil-to-lymphocyte ratio (NLR), monocyte-to-lymphocyte ratio (MLR), and PLR, following established veterinary methods. Serum C-reactive protein (CRP) concentrations were measured using a validated assay according to the manufacturer’s protocol. These biomarkers were selected to assess subclinical inflammatory status in cats with PKD.

### Statistical analysis

Data were assessed for normality before analysis and are presented as mean ± SEM. Continuous variables were compared using Student’s t-test or one-way analysis of variance, as appropriate. Pearson’s correlation analysis was used to evaluate associations among renal imaging parameters, genetic status, and inflammatory biomarkers. A p-value < 0.05 was considered statistically significant. Statistical analyses were performed using GraphPad Prism software (version 10.0) in accordance with standard biostatistical guidelines.

## RESULTS

### Clinical characteristics and baseline findings

All cats included in the study were clinically stable at the time of evaluation. None exhibited overt clinical signs commonly associated with advanced renal disease, including anorexia, weight loss, vomiting, polyuria, polydipsia, lethargy, or dehydration. Physical examination parameters, including body condition score, hydration status, heart rate, respiratory rate, and blood pressure, were within acceptable age- and breed-specific reference ranges. Importantly, no cats were classified as azotemic, and none fulfilled the diagnostic criteria for chronic kidney disease at enrollment. No mortality events occurred during the study period, and no cats required euthanasia or were lost to follow-up because of renal-related complications. As this was a retrospective, clinically oriented study involving client-owned animals, postmortem examination and histopathological evaluation were not available and were therefore not included in the analysis.

### Baseline characteristics of the study animals

The most prevalent breeds among the enrolled cats were Scottish Fold (n = 9, 32.14%), Persian (n = 7, 25%), domestic shorthair (n = 4, 14.28%), and mixed breeds (n = 8, 28.58%). Based on renal ultrasonographic findings and *PKD1* genotyping, cats were categorized into three groups: Group 1, healthy control cats without renal cysts and without *PKD1* mutation; Group 2, cats with polycystic kidneys and a wild-type *PKD1* genotype identified by ultrasonography; and Group 3, cats with polycystic kidneys carrying a heterozygous *PKD1* mutation. Group allocation and baseline characteristics are summarized in Table 1.

**Table 1:** Characteristics of the cats.

Parameters (Mean ± SEM)	Total (n = 28)	Group 1 (n = 17)	Group 2 (n = 6)	Group 3 (n = 5)	p-value
Age (years)	5.34 ± 2.25	5.32 ± 2.67	5.83 ± 0.69	4.80 ± 1.72	0.7711
Weight (kg)	3.50 ± 0.85	3.57 ± 1.01	3.20 ± 0.52	3.60 ± 0.28	0.6515
Male, n (%)	50	47	50	60	–
Cyst in both kidneys, n (%)	39.28	0	100	100	–

SEM = Standard error of the mean, n = Number of cats, – = Not applicable (no statistical comparison performed for categorical variables in this context).

### Ultrasonographic findings

Renal ultrasonographic findings for all cats are presented in Table 2. Cats in Groups 2 and 3 exhibited multiple, well-defined anechoic renal cysts with distal acoustic enhancement, consistent with polycystic kidney morphology. Cysts were bilaterally distributed in affected cats and varied in size and number. Significant differences in kidney length and height were observed among groups, reflecting structural renal remodeling in cats with cystic disease. In contrast, the kidney-to-aortic diameter ratio did not differ significantly among groups, indicating consistent normalization for body size across populations. Representative ultrasonographic images are shown in Figure 1.

**Table 2:** Ultrasonographic parameters.

Parameters (Mean ± SD)	Total (n = 28)	Group 1 (n = 17)	Group 2 (n = 6)	Group 3 (n = 5)	p-value
Average kidney length (cm)	4.04 ± 0.86	3.76 ± 0.45***	4.07 ± 0.59***	4.93 ± 1.39***	0.0001
Average kidney length/aorta	13.90 ± 2.42	13.89 ± 2.28	14.51 ± 2.12	13.85 ± 2.98	0.7977
Average kidney height (cm)	2.18 ± 0.48	2.02 ± 0.29*	2.06 ± 0.27	3.01 ± 0.49*	0.0271
Systolic blood pressure (mmHg)	137.64 ± 18.69	139.09 ± 16.62	141.72 ± 20.39	128.40 ± 19.92	0.4820

SD = Standard deviation, n = Number of cats,

* p < 0.05,

**p < 0.01,

*** p < 0.0001. A statistically significant difference was observed among groups using analysis of variance.

### Hematological and biochemical profiles

Hematological and biochemical parameters for all cats (n = 28) are summarized in Table 3. To minimize confounding effects, cats with pre-existing hematologic disorders, diagnosed chronic kidney disease, or a history of anticoagulant therapy were excluded before analysis. Mean concentrations of creatinine, blood urea nitrogen, and SDMA did not differ significantly among groups (p > 0.05), indicating preserved renal excretory function across all study populations. Similarly, total plasma protein and CRP concentrations showed no statistically significant intergroup differences.

**Table 3:** Blood profiles of all cats.

Parameters (Mean ± SD)	Total (n = 28)	Group 1 (n = 17)	Group 2 (n = 6)	Group 3 (n = 5)	p-value
Plasma Cr concentration (mg/dL)	1.26 ± 0.32	1.26 ± 0.27	1.46 ± 0.38	1.10 ± 0.32	0.1939
BUN (mg/dL)	21.33 ± 3.59	20.94 ± 3.59	21.13 ± 2.82	22.88 ± 1.78	0.5905
SDMA (μg/dL)	16.67 ± 2.11	17.21 ± 1.65	16.0 ± 1.29	15.20 ± 3.12	0.1458
TP (g/dL)	7.27 ± 0.49	7.19 ± 0.50	7.21 ± 0.39	7.63 ± 0.37	0.2025
NLR	4.68 ± 4.0	3.82 ± 1.77*	9.17 ± 8.13	4.09 ± 1.80*	0.0508
MLR	0.17 ± 0.11	0.17 ± 0.11	0.11 ± 0.05*	0.28 ± 0.13*	0.0534
PLR	166.14 ± 27.00	147.07 ± 17.00	155.97 ± 6.00	258.19 ± 4.00	0.3031
CRP (mg/dL)	0.13 ± 0.05	0.15 ± 0.05	0.12 ± 0.04	0.10 ± 0.01	0.0843

SD = Standard deviation, n = Number of cats, Cr = Creatinine, BUN = Blood urea nitrogen, SDMA = Symmetric dimethylarginine, TP = Total plasma protein, NLR = Neutrophil-to-lymphocyte ratio, MLR = Monocyte-to-lymphocyte ratio, PLR = Platelet-to-lymphocyte ratio, CRP = C-reactive protein,

* p < 0.05,

**p < 0.01, ***p < 0.0001. A statistically significant difference was observed among groups using analysis of variance.

In contrast, inflammatory cell ratios demonstrated notable trends. NLR tended to be higher in Group 2 (9.17 ± 8.13) compared with Group 1 (3.82 ± 1.77) and Group 3 (4.09 ± 1.80), approaching statistical significance (p = 0.0508). MLR showed a similar borderline difference (p = 0.0534), with higher values observed in Group 3 (0.28 ± 0.13). PLR was numerically elevated in Group 3 (258.19 ± 4.00), although this difference did not reach statistical significance.

### Correlation between inflammatory biomarkers and ultrasonographic parameters

**Figure 2:**
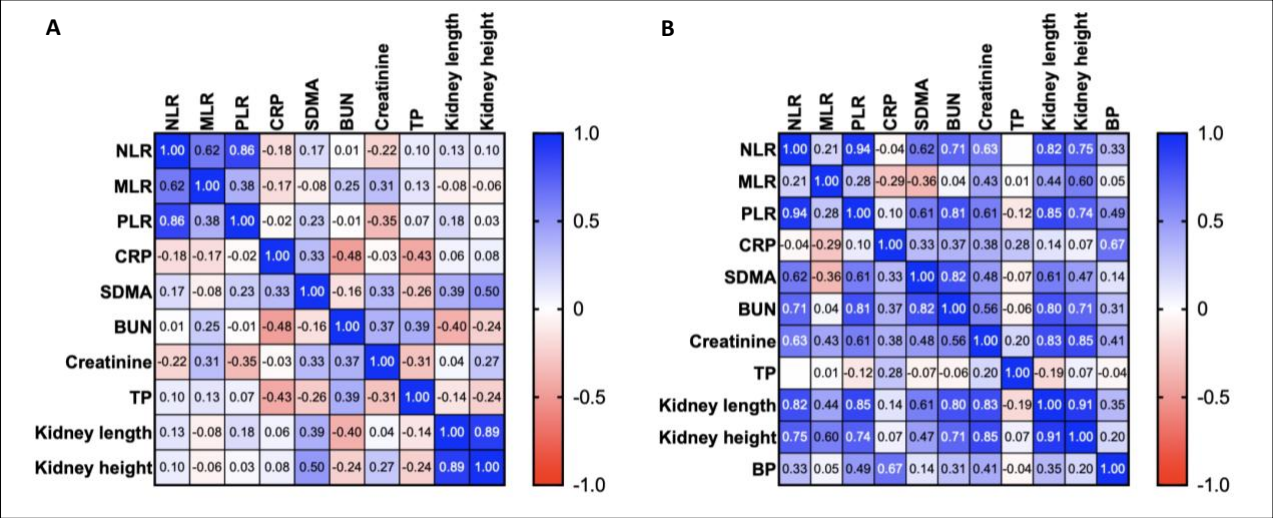
Heatmap of the Pearson correlation matrix showing relationships among hematological inflammatory ratios (NLR, MLR, and PLR), biochemical markers (CRP, SDMA, BUN, creatinine, and TP), and renal morphometric parameters (kidney length, kidney height). (A) Cats without renal cysts. (B) Cats with renal cysts.

**Figure 3:**
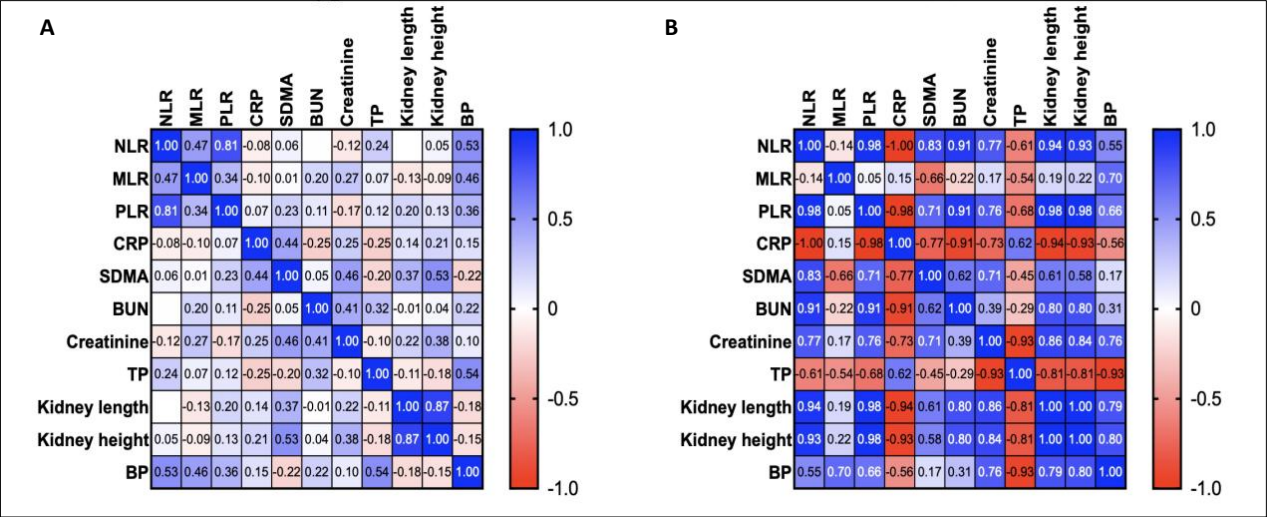
Correlation matrix of hematological inflammatory ratios (NLR, MLR, and PLR), biochemical markers (CRP, SDMA, BUN, creatinine, and TP), and *PKD1* genotype. (A) Cats with wild-type *PKD1* genotype. (B) Cats with a heterozygous *PKD1* mutation.

Correlation matrixes are illustrated in Figures 2 and 3. In clinically healthy cats (Group 1), moderate positive correlations were observed among inflammatory ratios, particularly between NLR and PLR (r = 0.86) and between NLR and MLR (r = 0.62). However, inflammatory biomarkers showed weak or negligible associations with renal biochemical indices and kidney morphometric measurements. In contrast, cats with renal cysts (Groups 2 and 3 combined) demonstrated markedly stronger correlations. NLR correlated strongly with PLR (r = 0.94) and showed positive associations with blood urea nitrogen (r = 0.71), creatinine (r = 0.63), SDMA (r = 0.62), and kidney morphometrics (length r = 0.82, height r = 0.75). PLR also correlated positively with blood urea nitrogen (r = 0.81) and creatinine (r = 0.61). These findings indicate an emerging link between low-grade systemic inflammation and structural renal changes in cats with cystic kidney disease, even in the absence of overt renal dysfunction.

### Pathological and molecular considerations

No gross pathological specimens, histopathological slides, or postmortem findings were available, as all cats were alive, clinically stable, and managed under standard clinical care. Consequently, renal histopathology and downstream molecular pathway analyses were beyond the scope of this retrospective study. Molecular investigation was limited to *PKD1* genotyping, and intracellular signaling pathways or molecular mechanisms related to cystogenesis were not assessed.

## DISCUSSION

### Overview of principal findings

This study examined the relationship between systemic inflammatory indices, renal structure, renal function, and *PKD1* genotype in cats with renal cystic disease. The principal finding is that systemic inflammatory activation is detectable in cats with cystic kidney changes despite preservation of conventional renal biomarkers, and that this inflammatory signal is more pronounced in cats carrying a *PKD1* mutation. These findings support the concept that inflammation precedes overt renal dysfunction in feline PKD and parallels structural renal remodeling.

### Inflammatory indices in early and subclinical PKD

Previous studies on feline PKD have focused largely on genetic diagnosis and imaging characteristics, with limited emphasis on systemic inflammatory responses during early disease stages. Consistent with earlier reports, cats in the present study showed normal creatinine, blood urea nitrogen, and SDMA concentrations, indicating preserved renal excretory function in early or compensated PKD [15, 16]. Similar observations have been reported in both feline and human PKD, where substantial nephron loss may occur before biochemical markers exceed reference ranges.

Despite stable renal biomarkers, NLR, MLR, and PLR demonstrated distinct group-related trends, suggesting early immune activation. The elevated NLR observed in cats with cystic kidneys and wild-type *PKD1* genotype may reflect acute or intermediate inflammatory responses associated with mechanical cyst expansion and epithelial injury. Comparable increases in NLR have been described in human autosomal dominant PKD and experimental renal cystic models, where neutrophil recruitment precedes interstitial fibrosis [17, 18].

In contrast, the higher MLR observed in cats carrying a *PKD1* mutation suggests a shift toward monocyte- and macrophage-driven chronic inflammation, a recognized feature of progressive PKD. Macrophage infiltration is a hallmark of cystic kidneys and contributes to fibroblast activation, extracellular matrix deposition, and cyst enlargement [19]. These findings indicate that distinct inflammatory profiles may characterize different stages of disease progression or genetic backgrounds in PKD.

### CRP and platelet-related responses

CRP concentrations did not differ significantly among groups, in agreement with previous feline studies reporting limited sensitivity of CRP in chronic, low-grade inflammatory conditions, particularly renal disease. Unlike acute inflammatory disorders, PKD progression is insidious, and systemic CRP responses may remain subdued until advanced stages.

The numerical increase in PLR observed in cats with *PKD1* mutations suggests a potential role for platelet activation in chronic renal inflammation. Platelets are increasingly recognized as active contributors to renal fibrogenesis through the release of pro-inflammatory and pro-fibrotic mediators, including platelet-derived growth factor and transforming growth factor-β [18, 19]. Although PLR differences did not reach statistical significance, this trend supports its possible utility as a supplementary indicator of disease activity.

### Relationship between inflammation, renal structure, and function

Correlation analyses demonstrated a progression from weak associations in healthy cats to strong, multivariate correlations in cats with cystic kidneys and *PKD1* mutations. In non-cystic cats, inflammatory and renal parameters were largely independent, reflecting physiological homeostasis. In contrast, cats with renal cysts exhibited strong positive correlations between inflammatory indices, kidney size, and renal biochemical markers, even though these values remained within reference ranges.

These findings parallel observations in human PKD, where kidney enlargement correlates more closely with disease progression than early biochemical markers [20, 21]. The strong associations between inflammatory indices and renal morphometrics in *PKD1*-mutated cats suggest that kidney enlargement may promote systemic inflammation through mechanisms such as tubular stretch, epithelial stress, and cytokine release.

### Genetic influence and molecular pathophysiology

The amplified correlations observed in cats carrying *PKD1* mutations underscore the central role of genetic disruption in PKD pathogenesis. The *PKD1* gene encodes polycystin-1, which is essential for renal tubular epithelial mechanosensation and calcium signaling. Loss of polycystin-1 function results in dysregulated intracellular calcium homeostasis, mitochondrial dysfunction, oxidative stress, and activation of inflammatory transcription factors, including NF-κB [22, 23].

The observed relationships between inflammatory markers, renal function indices, and blood pressure in *PKD1*-mutated cats support a model in which a systemic inflammatory–renal–vascular response is initiated by genetic mutation. Hypertension, which correlated with inflammatory and renal parameters in this study, is a recognized consequence of PKD progression and contributes to further nephron injury and cyst expansion [24]. Collectively, these findings indicate that systemic inflammation is an integral component of PKD pathophysiology rather than merely a secondary consequence of renal dysfunction.

### Clinical implications

The results suggest that hematological inflammatory ratios, particularly NLR and MLR, may serve as accessible and cost-effective tools for identifying early inflammatory activity in cats with renal cysts, even before the development of azotemia. Integration of these indices with ultrasonography and genetic testing may enhance risk stratification, disease monitoring, and early intervention strategies in feline PKD.

### Limitations

This study has several limitations. The sample size was relatively small, which may limit statistical power and generalizability. As a retrospective, single-center study, environmental influences, dietary factors, and subclinical comorbidities could not be fully controlled. Because all cats were alive and clinically stable, histopathological confirmation and postmortem evaluation were not available, precluding direct assessment of renal fibrosis, inflammatory infiltrates, and cyst wall pathology. Molecular analyses were limited to *PKD1* genotyping, and downstream signaling pathways involved in cystogenesis and inflammation were not directly evaluated. Finally, the duration of follow-up was limited. Longitudinal studies with larger cohorts, repeated inflammatory assessments, advanced imaging, and integration of molecular and histopathological data are needed to validate inflammatory ratios as prognostic biomarkers in feline PKD.

## CONCLUSION

This study demonstrated that systemic inflammatory activation is detectable in cats with renal cystic disease despite preserved conventional renal biomarkers, including creatinine, blood urea nitrogen, and SDMA. Inflammatory indices derived from routine hematology, particularly NLR and MLR, showed stronger associations with renal morphometric parameters and biochemical indices in cats with renal cysts than in clinically healthy controls. These associations were most pronounced in cats carrying a heterozygous *PKD1* mutation, indicating that genetic background amplifies the link between inflammation, renal structure, and early functional alterations. Notably, inflammatory biomarkers correlated with kidney size and blood pressure even when azotemia was absent, highlighting their sensitivity in subclinical PKD.

The findings suggest that simple hematological inflammatory ratios, such as NLR and MLR, may serve as accessible, low-cost adjunct tools for early detection and monitoring of PKD in cats. When used alongside ultrasonography and *PKD1* genotyping, these indices may improve clinical risk stratification, facilitate earlier intervention, and support longitudinal monitoring before overt renal dysfunction develops.

A key strength of this study is the integrated evaluation of systemic inflammation, renal morphometrics, biochemical markers, and *PKD1* genotype in clinically stable, non-azotemic cats. The use of readily available hematological indices enhances the translational relevance of the findings for routine clinical practice. In addition, the inclusion of genetic data allowed discrimination between cystic disease driven by structural changes alone and that influenced by underlying *PKD1* mutation.

Future studies should incorporate larger, multicenter cohorts with longitudinal follow-up to validate the prognostic value of inflammatory ratios in PKD. Integration of repeated inflammatory assessments, advanced imaging modalities, and histopathological and molecular analyses will be essential to clarify the temporal relationship between inflammation, cyst expansion, and renal dysfunction. Evaluating the response of inflammatory indices to therapeutic interventions may also provide insight into their utility as monitoring biomarkers.

Overall, this study supports the concept that systemic inflammation is an early and integral component of feline PKD, particularly in cats carrying *PKD1* mutations. Hematological inflammatory ratios offer promising complementary markers that may enhance early recognition and clinical management of PKD before irreversible renal damage occurs.

## DATA AVAILABILITY

All the generated data are included in the manuscript.

## AUTHORS’ CONTRIBUTIONS

SP: Identified the research topic and study area and drafted the manuscript. SP and KJ: Performed the study, developed the methodology, and conducted data analysis and interpretation. KJ, PJ, WM, and RB: Analyzed and interpreted the data and revised the manuscript. All authors have read, reviewed, and approved the final version of the manuscript.
